# Bristol Medico-Chirurgical Journal "The Medical Journal of the South West"

**Published:** 1988-02

**Authors:** 


### Bristol Medico-Chirurgical Journal
### "The Medical Journal of the South West"

### 'Clinical Press'

From the ashes of John Wright, once Britain's leading medical publisher, has arisen phoenix-like a new Bristol medical publisher, CLINICAL PRESS. It is born of a partnership between David Kingham, lately managing director of John Wright and Paul Goddard, clinical radiologist, author of textbooks of radiology and assistant editor of this journal; between them they possess great publishing expertise and the entrepreneurial spirit in a high degree. They intend to enter the territory vacated by John Wright and will start by publishing a series of popular textbooks aimed at the postgraduate student. They deserve to succeed and we wish them well. They have also undertaken to publish this journal for a year at least. Readers will remember that HERMISTON has published the journal during the last two years, initially with the sponsorship of Bristol-Myers at whose insistance we adopted the supplementary title of BRISTOL MEDICINE. After a year Bristol Myers dropped us, and we shall now drop the supplementary title, retaining the descriptive title THE MEDICAL JOURNAL OF THE SOUTH WEST. CLINICAL PRESS will continue to distribute the journal to doctors in the West of England without charge, hoping that a big circulation will attract the advertising revenue. Nevertheless the society will make a substantial contribution and attempts are being made to gain funding from other Bristol sources and to persuade them that this centenarian journal contributes to education and provides a service to medicine in the West of England. An ideal and a model which we can have in mind is the NEW ENGLAND JOURNAL OF MEDICINE based on Boston. The journal will have to carry more advertisements, some not strictly medical and we hope to improve the distribution. Hermiston from Edinburgh employed a medical mailing firm and we know there were omissions and duplications. CLINICAL PRESS will compile its own list and with local knowledge should do better.

### Crohn-Dalziel's disease.

*Mr Michael Harmer*
*Has donned his moral armour,*
*And started a campaign*
*To link Crohn's with Dalziel's name,*

On another page we publish an article on the renaming of a disease—Crohn's disease—by Mr Michael Harmer, a surgeon whose exceptional interest in the condition began when, as a medical student in 1936, he had a resection of the caecum and terminal ileum, two years after the 'landmark article' by Crohn, Ginzburg and Oppenheimer (1), and who remains in excellent health 51 years later (2). The surgeon, Mr Harold Wilson thought the condition was tuberculous but a subsequent histological diagnosis of terminal ileitis was made by Dr Robb Smith who had read Crohn's paper. The chance of this article being presented to our journal came about because in a paper in 1950 your editor expressed the opinion that Crohn's disease was well named (3), and so Mr Harmer wrote to him enquiring if, in the light of Ginzburg's recent writings, (4) he still held to his opinion. The disease which has come to be called Crohn's disease is not the 'terminal ileitis' described by Crohn et al. in 1934, it is, as Ginzburg pointed out, the 'chronic interstitial enteritis' described by Dalziel in 1913. Mr Harmer asked Dr Ginzburg, now in his 89th year, who has never used the Crohn eponym, his view on the nomenclature and he states firmly that it should be named Dalziel's disease. Mr Harmer wrote an article arguing this proposition but it was rejected by both our national weekly medical journals on the grounds that the proposal had been made before by Kyle (5) and that the matter is not very important anyway. However your editor is a convert to the proposition and believes that another shove may get the ball rolling, he is glad to provide Mr Harmer with a vehicle, however low power, and incidentally our readers with an interesting piece of history and a debate which some may feel moved to enter. A friend of Michael Harmer, the scholar/neurologist, Dr John Penman celebrated the 50th anniversary of his resection in Greek elegiac couplets and also provided the following translation:

*Since a yard or more was cut,*
*Michael, from your ailing gut,*
*You, defying Dr Crohn,*
*Have for 50 years lived on,*
*And survived him. Undismayed,*
*May you long defy his shade.*

1. CROHN, B. B., GINZBURG, L., OPPENHEIMER, G. D. (1932) *J.A.M.A.*, **99**, 1323–1329.
2. HARMER, M., BAILEY, A. G. S. (1986) *Lancet* 94.
3. ARMITAGE, G., WILSON, M. G., (1950) *Br.J.Surg.*, **38**, 192–193.
4. GINZBURG, L., *Gastroenterology* (1986) **90**, 1410.
5. KYLE, J., (1979) *B.M.J.* **1**, 876–877.

